# Matrix Metalloproteinases Are Differentially Regulated and Responsive to Compression Therapy in a Red Duroc Model of Hypertrophic Scar

**Published:** 2018-01-05

**Authors:** Taryn E. Travis, Pejhman Ghassemi, Nicholas J. Prindeze, Lauren T. Moffatt, Bonnie C. Carney, Abdulnaser Alkhalil, Jessica C. Ramella-Roman, Jeffrey W. Shupp

**Affiliations:** ^a^The Burn Center, Department of Surgery, MedStar Washington Hospital Center, Washington, DC; ^b^Firefighters’ Burn and Surgical Research Laboratory, MedStar Health Research Institute, Washington, DC; ^c^Department of Biomedical Engineering, Catholic University of America, Washington, DC; ^d^Department of Biomedical Engineering, Florida International University, Miami

**Keywords:** hypertrophic scar, pressure treatment, matrix metalloproteinase, extracellular matrix, wound healing

## Abstract

**Objective:** Proteins of the matrix metalloproteinases family play a vital role in extracellular matrix maintenance and basic physiological processes in tissue homeostasis. The function and activities of matrix metalloproteinases in response to compression therapies have yet to be defined. Here, a swine model of hypertrophic scar was used to profile the transcription of all known 26 matrix metalloproteinases in scars treated with a precise compression dose. **Methods:** Full-thickness excisional wounds were created. Wounds underwent healing and scar formation. A subset of scars underwent 2 weeks of compression therapy. Biopsy specimens were preserved, and microarrays, reverse transcription-polymerase chain reaction, Western blotting, and immunohistochemistry were performed to characterize the transcription and expression of various matrix metalloproteinase family members. **Results:** Microarray results showed that 13 of the known 26 matrix metalloproteinases were differentially transcribed in wounds relative to the preinjury skin. The predominant upregulation of these matrix metalloproteinases during early wound-healing stages declined gradually in later stages of wound healing. The use of compression therapy reduced this decline in 10 of the 13 differentially regulated matrix metalloproteinases. Further investigation of MMP7 using reverse transcription-polymerase chain reaction confirmed the effect of compression on transcript levels. Assessment of MMP7 at the protein level using Western blotting and immunohistochemistry was concordant. **Conclusions:** In a swine model of hypertrophic scar, the application of compression to hypertrophic scar attenuated a trend of decreasing levels of matrix metalloproteinases during the process of hypertrophic wound healing, including MMP7, whose enzyme regulation was confirmed at the protein level.

Matrix metalloproteinases (MMPs) influence cell responses to their microenvironment by activation of latent membrane proteins and reorganization of extracellular matrix (ECM) components by specific digestion. The activities of MMPs are vital to the mechanisms of wound healing such as cell migration, differentiation, survival, and proliferation. Thus far, 26 enzymes have been classified as members of the MMP family based on structural similarities, enzymatic activity, and dependency on metals such as calcium or zinc for their function. Varying substrates and localizations of MMPs in tissue were distinguished and considered in MMP classification as well.^1^ Specific inhibitors of MMPs include a group of 4 tissue inhibitor metalloproteinases (TIMP1-4)^2^ and the GPI-anchored glycoprotein Reversion-inducing cysteine-rich with Kazal motifs (RECK).^2^^,^^3^

MMPs are cleared by endocytosis after forming a thrombospondin-2 (TSP2)-dependent complex with low-density lipoprotein receptor-related protein (LDL-RP)^4^ or after forming an α_2_-macroglobulin/MMP complex.^5^ Deregulation of MMPs or their inhibitors has been linked to the failure of arterial bypass grafts,^6^ asthma,^7^ cardiovascular disease,^8^ metastasis,^9^ and all different stages of tumorigenesis.^10^ MMPs are known to degrade basement membranes and matrix components through the activation of chemokines and growth factors, leading to their importance in many physiological and pathophysiological processes.

The red Duroc pig is an acceptable model to study wound healing and hypertrophic scar formation due to its similarities to human skin at the gross, cellular, and molecular levels.^11^^-^14 With the creation of a full-thickness excisional wound, this animal provides a reliable, validated model of fibroproliferative scarring, which allows for investigation of the normal and aberrant physiological processes of human skin wound healing.^15^

The well-documented burden of hypertrophic scar following cutaneous trauma^16^^-^18 continues to demand investigation into its pathophysiology in order to identify therapeutic targets. Compression therapy, a widely employed method for the prevention and treatment of hypertrophic scar,^19^ is as yet poorly understood and difficult to standardize among patients for scientific study. The work presented here aimed to use a validated animal model to test standardizable compression therapy in order to investigate whether MMP expression differs as a result of the application of compression to scar. Matrilysin or MMP7, the smallest known MMP (molecular weight: 28 kD), has been the focal point in many acute mucosal epithelial injury studies. Studies include those pertaining to infection, inflammation, and epithelial migration in the airway^20^^,^^21^ and gastrointestinal tract,^22^ as well as the peptide's involvement in tumorigenesis and metastasis. Despite the known role of MMP7 in fibrotic processes in multiple organs, which mimic those described in dermal and epidermal wound-healing and scar formation,^23^ the extent of involvement of MMP7 in scar development, especially during and after compression therapy, remains unknown. MMP7 is known to specifically degrade elastin and to respond to mechanical stimuli in other systems.^24^ For these reasons, MMP7 was the focus of this work.

## MATERIALS AND METHODS

### Animal model and wound creation

All animal work described was reviewed and approved by the MedStar Health Research Institute's Institutional Animal Care and Use Committee. Juvenile castrated male Duroc swine (30-55 kg) (n = 2) were received and handled according to facility standard operating procedures under an animal care and use program accredited by the Association for Assessment and Accreditation of Laboratory Animal Care International and Public Health Service Animal Welfare assured.

**Figure 1:**
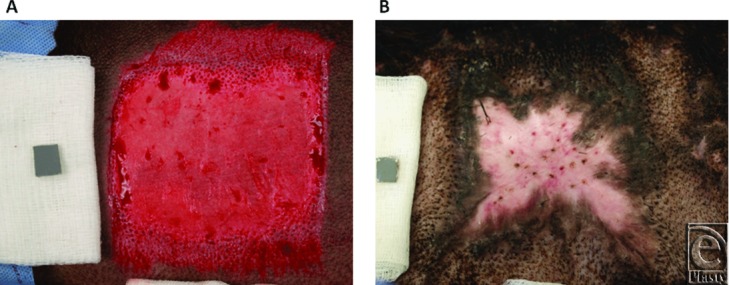
Representative images of full-thickness excisional wounds (a) and resulting fibroproliferative scars (b) in the Duroc pig model.

On the day of surgery, animals were anesthetized with a combination of ketamine and xylazine delivered intramuscularly. Animals were intubated, maintained on isoflurane, placed on a warming blanket, and ventilated while heart rate, blood pressure, peripheral oxygen saturation, end-tidal carbon dioxide saturation, and core body temperature were continuously monitored by trained research staff. Body hair was clipped, and the skin was prepped with chlorhexidine gluconate scrub. To create full-thickness wounds, one 4 × 4-in (10.16 × 10.16-cm) square was excised over the rib cage on each side of the animal, at a total depth of 0.090 in (0.030 in × 3 passes) using a Zimmer dermatome (Zimmer, Ltd, Swindon, United Kingdom) (Fig 1*a*). In previous experience, this wound creation technique consistently results in hypertrophic scars^15^ (Fig 1*b*). Punch biopsy specimens (3 mm) were taken at baseline (preexcision) and immediately postexcision and placed in either formalin for subsequent histology or in Allprotect Tissue Reagent (Qiagen, Valencia, Calif) for subsequent RNA and protein isolation. Mepilex Ag dressings (Monlylke, Gothenburg, Sweden) were applied to wounds after wound creation and at each dressing change. Animals were fitted with custom-made 2- to 3-mm thick neoprene vests,^25^ which completely covered the applied dressings while allowing mobility of the animal. Buprenorphine and fentanyl were administered for pain control at the end of each surgical procedure. Animals returned to the operating room every 7 days until day 133 postwound creation for examinations, imaging, dressing changes, and biopsies. The animals were examined at least twice daily to monitor animal health and to identify any signs of pain, wound infection, or distress.

### Compression delivery system

**Figure 2:**
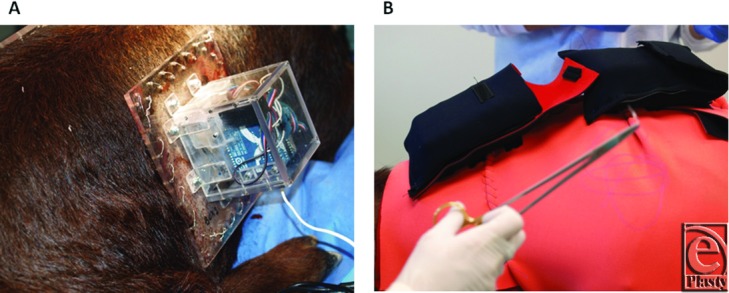
Example images of the pressure delivery device mounted over the hypertrophic scar at day 70 (a) and the outer neoprene vest with neoprene battery pack pouches (b).

A compression device was created to deliver a precise level of pressure as previously described^26^ (Fig 2*a*). Briefly, the device consists of a set of linear actuators connected to an aluminum plate and enclosed in a robust Plexiglas box. The actuators are attached to the corners of the box and set the aluminum plate to predetermined positions to allow for delivery of pressure to the underlying tissue. Local pressure measurements are obtained with a low-profile pressure sensor. The sensor is connected to a microcontroller board programmed to maintain a desired pressure level. A wireless communication board is connected to the microcontroller within the box, allowing for wireless communication to a laptop computer for recording of pressure data and controlling of the linear actuators.

To apply the devices to hypertrophic scars, the device is secured to the surrounding skin with MYO/WIRE II Sternotomy Suture (A&E Medical Corporation, Durham, NC) and then covered with a neoprene vest with a cutout to allow the pressure delivery box to protrude. Lightweight gauze is used to build a protective dome of padding around the boxes and dorsum of the animal prior to application of Delta fiberglass casting material (BSN Medical, Charlotte, NC) to reinforce this dome. An additional outer neoprene vest is finally custom-fitted over the entire apparatus to ensure protection of the animal as well as pressure delivery and data-streaming devices. Two battery packs to power the devices are mounted in neoprene holsters on the dorsum of the animal attached to the outer vest (Fig 2*b*).

For 2 weeks, starting at day 70 postwound creation, 30 mm Hg of pressure was applied to scars with the pressure delivery devices. At the midpoint of the 2 weeks, animals returned to the operating room for clinical evaluation of their scars, imaging, and biopsies. Of the 2 scars per animal, one served as a treated scar and one served as a sham, which held a pressure box without active pressure delivery.

### Microarray performance

A total of 28 biopsy specimens were collected from 2 red Duroc pigs and used in the evaluation of genome-wide transcriptome changes during the course of wound healing and compression application. Four biopsy specimens were collected from the left and right flanks of 2 pigs before wound creation and were used as a baseline of gene transcription. For assessment of wound progression and compression effects on scar during early, mid, and late wound-healing stages, 8 biopsy specimens were harvested on day 49, days 91-99, and days 119-133, respectively (4 per animal, 2 compression treated and 2 sham). Compression was applied between days 70 and 84 as indicated earlier. mRNA isolation is described in the “RNA and protein isolation” section.

Microarray assays were performed using Agilent-026440 S. scrofa (Pig) Oligo Microarray v2 expression array (GE 4x44K v2 2-color microarray) slides and kits (Agilent Technologies, Inc, Santa Clara, Calif) following the manufacturer's protocol. Hybridized microarray slides were scanned using Agilent Technologies Scanner G2505C US09493743.

Images of scanned microarray slides were feature-extracted and normalized using Agilent's feature extraction software, version 10.7 or later, in the default setup (Agilent Technologies, Inc).

### Immunofluorescence

Punch biopsy specimens taken from areas of wound and scar were fixed in 10% formalin and embedded in paraffin. Paraffin blocks were sectioned, 3 sections per slide, at a thickness of 5 μm and baked at 60°C. After cooling, slides were deparaffinized and rehydrated with PBS. Antigen retrieval was performed in Tris-EDTA (ethylenediaminetetraacetic) buffer at 95°C to 100°C for 20 minutes. Slides were then cooled for 5 minutes in running cold water. Slides were washed in 0.025% Triton X-100 for 5 minutes × 2. Slides were blocked in 5% nonfat milk, 1% BSA, in PBS for 1 hour. After blocking, slides were incubated with polyclonal rabbit anti-MMP7 (Abcam, Cambridge, United Kingdom) primary antibody diluted in PBS-0.05% Tween 20 at 4°C overnight. Negative control slides were incubated only with PBS-Tween 20. After overnight incubation with primary antibody, slides were rinsed with 0.025% Triton X-100 for 5 minutes × 2. Slides were then treated with polyclonal goat anti-rabbit IgG-CY3–conjugated (Abcam) secondary antibody diluted in PBS-0.05% Tween 20 for 1 hour at room temperature. Slides were rinsed with PBS for 5 minutes × 3 and then counterstained with DAPI (Santa Cruz Biotechnology, Dallas, Tex) for 10 minutes. Slides were then viewed with a Zeiss Axioimager microscope and a multichannel black and white camera equipped with fluorescence filters (Carl Zeiss, Oberkochen, Germany). Zeiss Zen software (Carl Zeiss) was used to quantify CY3 expression colocalized with DAPI expression in evaluated samples, and this was normalized to the same expression in the baseline uninjured skin. MMP7 protein expression was quantified by immunofluorescence in one of the 2 animals. One high-powered field per section, with 3 sections per slide for each scar, was evaluated. Before treatment began, only 1 scar was evaluated and after treatment, the treated and sham scars were evaluated.

### RNA and protein isolation

Allprotect-preserved biopsy specimens were subjected to RNA and protein isolation according to the protocol outlined by Berglund et al.^27^ Tissue was homogenized in guanidinium thiocyanate (GITC) lysis buffer (5.1 M GITC, 50 mM sodium citrate, 50 mM EDTA, 0.5% β-mercaptoethanol [add just before use], and 0.5% *n*-lauroylsarcosine [add just after lysis]). Samples were then centrifuged at 14,000 *g* for 5 minutes at 4°C, and the supernatant was collected and added to 1 mL of phenol-chloroform. Next, 50 μL of 10% *n*-lauroylsarcosine and 100 μL of chloroform were added to the lysate and samples were mixed and incubated at room temperature for 5 minutes. Samples were centrifuged for 20 minutes at 14,000 *g* at 4°C. The aqueous layer was removed and added to a tube with 1 mL of phenol-chloroform and 100 μL of chloroform. The interphase layer and the lower organic phase were kept for DNA and protein isolation as explained later. For RNA purification, samples were centrifuged for 20 minutes as described earlier. The aqueous layer was removed, and 1 mL of isopropanol and 200 μL of 3 M sodium acetate were added. Samples were incubated at −20°C overnight. After overnight incubation, the samples were spun as described earlier for 20 minutes, the supernatant was removed, and the pellet was dissolved in 100 μL of RNase-free water. The RNEasy kit (Qiagen) was then used, starting with adding 350 μL of RNEasy RLT buffer. The manufacturer's protocol was followed for RNA extraction. RNA sample quality and quantity were assessed using a Bioanalyzer RNA 6000 NanoKit (Agilent Technologies, Inc) and recorded.

DNA (interphase of the aqueous and organic phases in the tube as described earlier) was removed by the addition of 100% ethanol (300 μL/1 mL). The samples were spun at 3000 *g* for 5 minutes at 4°C and the supernatant was transferred to 2 fresh tubes, where 1.5 mL of isopropanol was added to each tube and the samples precipitated overnight at −20°C. After overnight incubation, samples were spun at 12,000 *g* for 10 minutes at 4°C and pellets were visible. The pellets were washed by 2 rounds of incubation with 0.3 M guanidine hydrochloride in 95% ethanol for 20 minutes and centrifugation at 7500 *g* for 5 minutes at 4°C and discarding the supernatant. The pellets were then washed with 95% ethanol, air-dried, and then resuspended in 100 μL of resolubilization buffer for 20 minutes at 55°C (8 M urea, 10 mM DTT, 10 μL/mL proteinase inhibitor cocktail) (Sigma Aldrich, St Louis, Mo). Total protein samples were quantified according to the manufacturer's protocol for Coomassie Plus Protein Assay Reagent (Thermo Fisher Scientific Inc, Waltham, Mass).

Proteins of interest (MMP7) were isolated from total protein using Dynabeads Protein G (Thermo Fisher Scientific Inc) for immunoprecipitation according to the manufacturer's protocol. Rabbit polyclonal anti-MMP7 antibody (Abcam) was used with bis[sulfosuccinimidyl]suberate (Thermo Fisher Scientific Inc) as a cross-linker. A total of 10 μg (5 μg from each animal) from sham- or compression-treated scars was used in immunoprecipitation of MMP7.

### Real-time RT-PCR

Initially, the transcript of various genes of interest in wound healing was quantified in a subset of scar samples (Allprotect-preserved biopsy specimens from days 70, 77, 84, 90, and 98) using a multiplex real-time reverse transcription-polymerase chain reaction (RT-PCR) system (SABiosciences, Qiagen, Valencia, Calif). Briefly, RNA was isolated and handled as described later. First-strand cDNA synthesis was carried out using 100 ng of total RNA in an RT2 first-strand kit (SABiosciences, Qiagen) according to the manufacturer's instructions.

Plates with wells containing gene-specific primers and RT2 real-time SyBR Green/ROX PCR mix were purchased from SABiosciences and used according to the manufacturer's instructions for gene expression analysis. Assays were performed on an ABI Prism 7500Fast PCR system (Applied Biosystems, Foster City, Calif). A set of 5 reference genes was included in the analysis for each sample and used for normalization. The ΔΔC_t_ method^28^ was used for analyzing the resulting raw gene expression data as described later. Graphs and statistical analysis were performed using Prism GraphPad 6.0 (GraphPad Software, La Jolla, Calif). Statistical significance was set at *P* < .05.

On the basis of the results found in these initial assays, confirmatory real-time RT-PCR was performed on additional samples specifically investigating transcript levels of MMP7. RNA samples were diluted to 1 ng/μL and added to BioRad SyBR Green master mix (BioRad Laboratories Inc, Hercules, Calif) with gene-specific primers and reverse transcriptase in 96-well plates. As a reference gene, levels of porcine 18s rRNA were quantified in parallel with target genes. The following sequences (Integrated DNA Technologies, Coralville, Iowa) were utilized in PCR: Pig 18s rRNA, forward 5′-CCGCGGTTCTATTTTGTTGGTTTT-3′ and reverse 5′-CGGGCCGGGTGAGGTTTC-3′ and Pig MMP7, forward 5′-AACACTGGTCTGATGGTGGC-3′ and reverse 5′-TCAGACGAATGAGCCAGACC-3′, created using NCBI/Primer-BLAST with a product length of 94. Samples were loaded in a BioRad CFX96 RT system C1000 thermal cycler (BioRad Laboratories Inc) and cycled as follows: step 1—50°C for 10 minutes; step 2—95°C for 5 minutes; step 3—95°C for 10 seconds; step 4—60°C for 30 seconds (repeat steps 2-4 for 39 cycles), followed by 95°C for 1 minute; step 5—55°C for 1 minute. The ∆∆*C*_t_ method was used in analyzing the resulting raw gene expression data.^28^ Briefly, the relative gene expression of the genes was calculated as ∆*C*_t_ sample = (*C*_t_ sample gene) − (*C*_t_ sample reference gene) and ∆*C*_t_ control = (*C*_t_ control gene) − (*C*_t_ reference gene).^28^ The fold regulation was then calculated as 2^-[Δ *C*_t_sample - Δ *C*_t_control]^, where control was represented by samples collected preexcision.

### Immunoblotting

Immunoprecipitated proteins were subjected to polyacrylamide gel electrophoresis using precast gels (BioRad Laboratories Inc) and a standard preparation Laemmli buffer along with Precision Plus Protein WesternC Standards (BioRad Laboratories Inc). Gel was transferred overnight to nitrocellulose membrane, followed by blocking with 5% nonfat milk, 1% BSA, in PBS for 1 hour. The membrane was then probed with polyclonal rabbit anti-MMP7 (Abcam; Ab38999) primary antibody diluted in PBS-0.05% Tween 20 at 4°C overnight. Polyclonal donkey anti-rabbit IgG-HRP (Abcam) was used as a secondary antibody, along with Precision Protein StrepTactin-HRP Conjugate (BioRad Laboratories Inc) for 1 hour at room temperature. After washing of the membrane, SuperSignal West Dura Extended Duration Substrate (Thermo Fisher Scientific Inc) was used for chemiluminescent detection of probed proteins, visualized with a Kodak Gel Logic 100 system (Eastman Kodak, Rochester, NY). Blot band strengths were quantified using Kodak Molecular Imaging Software (Eastman Kodak) and normalized to baseline uninjured tissue protein levels.

### Statistical analysis

A Grubb's outlier test was used on all values prior to calculation of averages, standard deviations, and statistical significance. A 2-tailed unpaired Student's *t* test was used to compare averages with statistical significance set at *P* < .05. Analysis and graphs were done using GraphPad Prism 6 (GraphPad Software).

## RESULTS

### MMPs transcription levels are modulated during wound healing and responsive to compression therapy

The microarray allowed for interrogation of the transcription levels of membrane-metalloendoproteinase (MME) and MMP1-3, 7-13, 15-17, 19-21, 23B, 24-28 representing 22 of the known 26 MMPs and 5 MMP inhibitors including TIMP1-4 and the α_2_-macroglobulin (A2M) gene. More than half of the MMPs (13/22) showed significant (fold change > 1.3 and/or *P* < .05) modulations in transcription levels in 1 or more designated biopsy collection stages. The predominant trend was upregulation, where 9 of the 13 MMPs were consistently upregulated (Table 1). Of the downregulated MMPs, MMP7 was by far the most downregulated MMP in the early, mid, and late wound-healing stages of sham and compression-treated wounds. Milder downregulation was observed in MMP25, and dual directional regulation was observed in MMP1 and to a lesser degree in MMP3 (Table 1). The application of 30 mm Hg compression for 14 days (biopsies from days 70-84 postinjury) increased the transcription levels of 10 of the 13 significantly modulated MMPs in the mid-stage biopsies (days 91-99). MMP9 was mildly further downregulated, and MMP3 and 14 were almost unchanged during the same period. The effect of compression persisted during the late-stage biopsies (days 119-133) in MMP7, 16, 19, and 25 (8 weeks postcompression) (Table 1). Two endogenous tissue inhibitors of metalloproteinases, TIMP1 and TIMP2, showed significant upregulation during early phase of wound healing that tended to decline during the course of wound healing and scar formation (Table 1). Application of compression seemed to ameliorate this upregulation, especially during later periods of wound healing and scar formation. Similarly, the MMP inhibitor A2M shows milder upregulation that tended to be reduced by compression.

### Multiplex real-time PCR confirms MMP7 expression seen in microarray data

**Figure 3:**
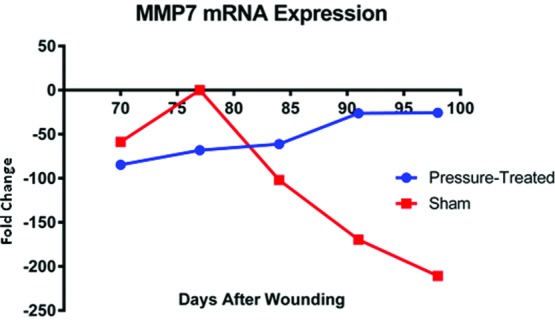
Multiplex real-time RT-PCR showing differential expression of MMP7. Compared with the uninjured skin, MMP7 mRNA was downregulated in the scar, and this downregulation was more pronounced in sham scars after the application of compression at days 84, 91, and 98. RT-PCR indicates reverse transcription-polymerase chain reaction.

In the microarray study of fibroproliferative wound healing and the effects of compression therapy on scar, MMP7 showed one of the more interesting transcription profiles. The transcription of this gene was studied independently at higher resolution using a multiplex RT-PCR and weekly biopsy specimens collected from day 70, the beginning point of 2 weeks of compression therapy and up to 3 weeks after the cessation of compression therapy. Results showed a transcription profile consistent with the transcription trend observed in microarrays. Compared with the uninjured skin, MMP7 mRNA was downregulated in sham-treated scar biopsies, and this downregulation was ameliorated after compression application at days 84, 91, and 98 (Fig 3). These initial effects were singled out for further investigation.

### Trends of MMP7 transcription levels in nontreated and compression-treated scars were reproduced using RT-PCR of additional biopsies

**Figure 4:**
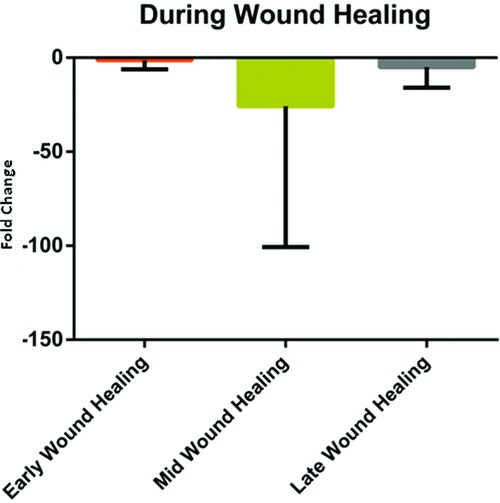
MMP7 confirmatory real-time RT-PCR showing transcript levels of MMP7 during early, mid, and late stages of wound healing relative to the uninjured skin. Error bars represent standard deviations of the mean. RT-PCR indicates reverse transcription-polymerase chain reaction.

Further confirmatory real-time RT-PCR was conducted on additional samples with a different set of primers to MMP7. These were again divided up into early, mid, and late phases of wound healing prior to the application of compression. Overall, transcript levels of MMP7 were decreased when compared with the baseline uninjured skin. The early phase of wound healing showed a 1.3 ± 4.9-fold downregulation of MMP7 expression from baseline. MMP7 was likewise downregulated in mid and late phases of wound healing by 25.8 ± 74.9-fold and 5.1 ± 10.8-fold, respectively (Fig 4).

**Figure 5:**
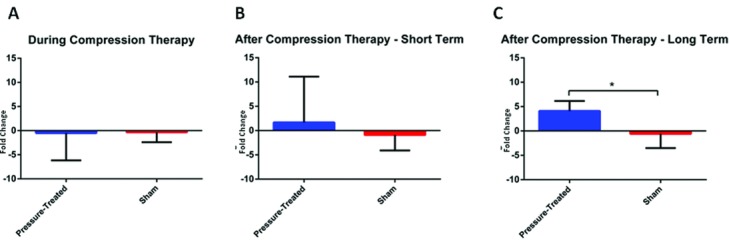
MMP7 confirmatory real-time RT-PCR showing transcript levels of MMP7 during the application of compression (a) and changes in these levels in the short term after compression therapy (b) and in the long term after compression therapy (c). Error bars represent standard deviations of the mean. *Denotes significant difference (*P* < .05). RT-PCR indicates reverse transcription-polymerase chain reaction.

Additional samples taken during compression therapy (days 77 and 84), shortly after compression therapy (days 91 and 98), and longer term after compression therapy (days 105-133) were subjected to the same real-time RT-PCR. In agreement with the previous results from microarray and multiplex RT-PCR, transcript levels of MMP7 were decreased compared with baseline during the course of compression therapy. Compression-treated scars showed a 0.5 ± 5.7-fold downregulation, whereas sham scars showed 0.3 ± 2.1-fold downregulation of MMP7 mRNA (Fig 5*a*). In the 2 weeks immediately following compression therapy, compression-treated scars showed an increase in MMP7 transcription with 1.7 ± 9.4-fold change whereas sham scars still showed generally decreased transcription of −0.9 ± 3.2-fold (Fig 5*b*). In the later weeks following compression therapy, from days 105 to 133, compression-treated scars showed a statistically significant increase in MMP7 mRNA expression compared with sham scars with 4.1 ± 2.1 versus −0.6 ± 2.9-fold regulations, respectively (*P* = .0003) (Fig 5*c*).

### Immunohistochemistry confirms observed trends of MMP7 transcription and compression-mediated modulations at protein levels

**Figure 6:**
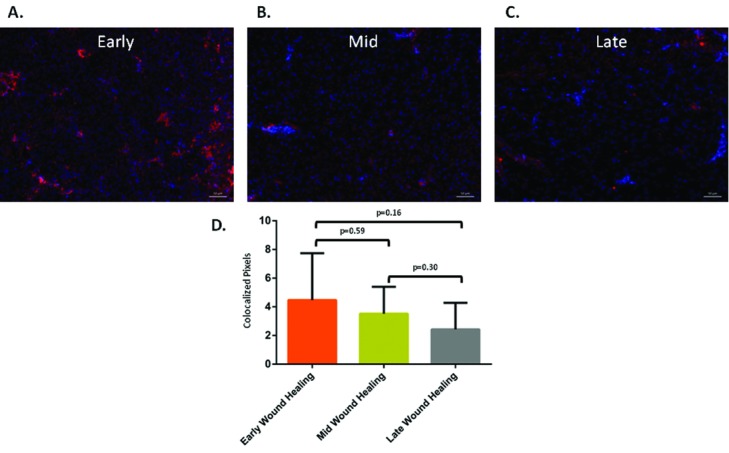
Representative images of sections from red Duroc pig skin scar biopsy specimens showing MMP7 immunofluorescence during early- (a), mid- (b), and late- (c) stage wound healing. Quantitative assessment of MMP7 expression using immunofluorescence results (d). Error bars represent standard deviations of the mean.

Biopsy specimens from early (days 7-21), mid (days 28-42), and late (days 49-70) phases of wound healing prior to compression delivery were subjected to immunofluorescence for the expression of MMP7. When normalized to the uninjured skin, biopsies showed the same decreasing trend of MMP7 transcription. The expression of MMP7 at early periods in wound healing (4.5 ± 3.3 pixels) (Fig 6*a*) was the largest, showing the highest levels of fluorescence, and this was followed by a trend of decreasing expression over time. Mid-phase wound healing showed 3.5 ± 1.9 pixels of colocalization (Fig 6*b*). Likewise, 2.4 ± 1.9 pixels were seen during late phase of wound healing (Fig 6*c*). Random software-aided measurements of fluorescence showed a congruent trend, although the differences did not reach statistical significance, and was confounded by the large standard deviations due to clustered distribution of fluorescence in histological specimens (*P* values of .59, .30, and .16; see Fig 6).

**Figure 7:**
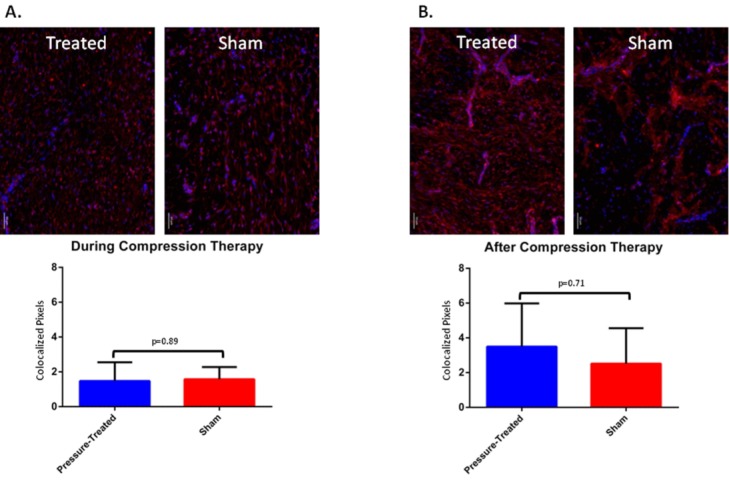
Representative images of sections from red Duroc pig scar biopsy specimens and quantitative assessment of MMP7 immunofluorescence during (a) and after (b) compression therapy. Error bars represent standard deviations of the mean.

Additional biopsy specimens taken during compression therapy (days 77 and 84) and after compression therapy (days 91-133) were subjected to the same evaluation. Qualitative assessment of scar sections stained for MMP7 showed comparable expression levels during compression therapy, with notable increases in MMP7 in compression-treated scar sections during the weeks following therapy. Quantitative fluorescence-based evaluation of MMP7 expression levels after normalization to baseline showed similar results in compression-treated and sham scars during the 2 weeks of compression therapy (1.5 ± 1.1 and 1.6 ± 0.7 pixels, respectively) (Fig 7*a*). After 2 weeks of compression therapy, there was a trend toward increased expression of MMP7 in compression-treated scars versus sham scars (3.4 ± 2.5 and 2.7 ± 1.9 pixels, respectively). This increase did not reach statistical significance due again to uneven fluorescence distribution and large standard deviation (Fig 7*b*) (*P* values of .89 and .71; see Fig 7).

### Western blots confirm the effects of compression therapy on MMP7 dowregulation

**Figure 8:**
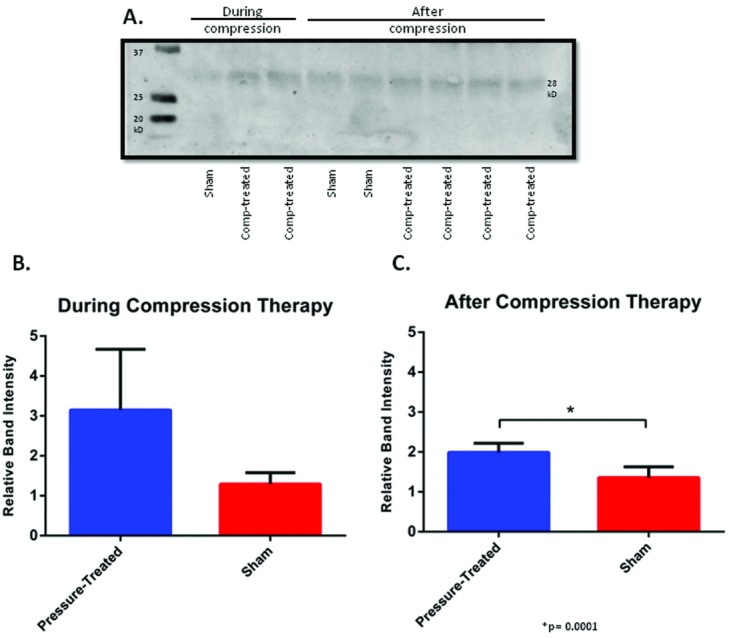
Western immunoblots showing overall protein levels MMP7 during and after compression therapy (a). Quantitative measurement of protein MMP7 inferred from Western blots during (b) and after (c) compression therapy. Error bars represent standard deviations of the mean.

Compression-treated and sham scar samples were obtained during and after compression therapy. Total protein was isolated and further processed to immunoprecipitate MMP7. Precipitated protein was subjected to SDS-polyacrylamide gel electrophoresis and Western blotting (Fig 8*a*). Bands from multiple blots were analyzed for intensity and normalized to the baseline uninjured skin. Overall, protein levels of MMP7 were low in all scar samples. When compression-treated and sham scars were compared, compression-treated scars showed a relative band intensity of 3.1 ± 0.8 versus sham scars with 1.3 ± 0.1 during the period of compression therapy, although this was not statistically significant with a *P* value of .0539 (Fig 8*a*, lower panel). In the weeks following compression therapy, the difference in MMP7 protein levels between compression-treated and sham scars did reach statistical significance (*P* = .0001), with compression-treated scars showing a relative band intensity of 2.0 ± 0.1 and sham scars showing 1.4 ± 0.1 (Fig 8*b*, lower panel).

## DISCUSSION

Tissue regeneration is essential to organ function and homeostasis. Balancing ECM breakdown and deposition is controlled by the activity of the MMP family.^29^ So far, 26 MMP enzymes sharing sequence, substrate specificity, and dependency on metals for their activity have been identified. The involvement of these enzymes in processes such as extravasation, cell migration, cell proliferation, apoptosis, and inflammation^30^^,^^31^ made them common identifiers of wound-healing outcomes^32^^,^^33^ and other pathological conditions such as cancer, metastasis, and rheumatoid arthritis.^31^ Here, we demonstrated using genome-wide transcriptomics that the majority of MMPs showed an increase in transcription during the course of wound healing. This makes sense in an environment of cell migration to populate a healing wound bed, neogenesis to support cell proliferation, and new ECM synthesis and reorganization. The results showed that MMP2, 9, 11, 12, 14-16, 19, 23B were steadily upregulated in wounds and scars throughout the study duration (up to 133 days). Downregulation was observed in MMP3, 7, and 25 in wound and scar tissues during the same duration. MMP1, which is known to be involved in blood coagulation^34^ and cellular response to drugs and fluid shear,^35^ among other activities, showed mild changes that included inversion of regulation. The changes seen may be explained by trauma to healing wounds due to animal movement and frequent biopsies. Similarly, interference from anesthetics and analgesics administrated to the animal cannot be excluded.

Posttranscriptional regulation of MMPs is controlled both by extracellular and membrane-bound proteases and by plasmin, whose role is to process inactive zymogen forms of MMPs to active forms. MMPs are also downregulated by the members of the MMP inhibitor family TIMP1-4 and A2M. Results from the transcriptomic work showed an increase in transcripts of TIMP1, 2 in scar tissue, suggesting some level of reduction in MMP activities despite higher transcription. Available information indicates that TIMP1 and 2 bind and regulate MMP1-3, 9, 14. Interactions with other MMPs are also possible, making it difficult to predict the net activity of all MMPs in scar under conditions of compression therapy. The observed mild and differential regulation of A2M seems to be consistent with the inflammatory environment of wounds and the cytokine transporter properties of this molecule rather than its inhibitory function.^36^ In general, the application of compression resulted in further upregulation in most of the MMPs and amelioration of the intensity of observed downregulation of MMP3 and 7. Compression had similar long-term effects on TIMP1, 2.

MMP7 has been shown to be responsive to mechanical stimulation in rat alveolar epithelial cells.^24^ Unlike other MMPs, MMP7 lacks a conserved C-terminal protein domain and has been thought to be absent in mature epidermis but is known to be present in the glandular elements of skin.^36^ The enzyme is known to degrade elastin, proteoglycans, type IV collagen, and fibronectin^36^ and to cleave α_1_-antitrypsin.^37^ Results of the present study showed that MMP7 is ubiquitously present at the mRNA and protein levels in both injured and uninjured red Duroc skin and is the most downregulated MMP in hypertrophic scar tissue. Compression application alleviated this observed downregulation of MMP7, which may be associated with detected improvements in scar treated with compression therapy.^38^^,^^39^

Overall conclusions include that the application of 2 weeks of 30 mm Hg of compression therapy to hypertrophic scars in Duroc pigs does exert differential expression of MMPs on both the transcript and protein levels. MMP7 does appear to be present in epidermal wounds and scars, albeit at a low level, and these levels appear to increase with the application of compression. Finally, these results allow for further investigation into the multitude of pathways that contribute to scar formation and remodeling to pave the way for a clearer understanding of how compression therapy works and additional possible therapeutic targets.

## Figures and Tables

**Table 1:** Differentially regulated matrix metalloproteinases and their inhibitors using genome-wide transcription profile of scar biopsies with or without 14-day compression treatment*

	Sham						Compression treated					
	Wound and scar		2 wk of device with no pressure				Wound and scar		2 wk device/30 mm Hg pressure, device removed			
	Early		Mid		Late		Early		Mid		Late	
Metallo-proteases/inhibitor type	log FC	*P*	log FC	*P*	log FC	*P*	log FC	*P*	log FC	*P*	log FC	*P*
**MME**	−0.109	7.70E-01	−0.205	6.38E-01	−1.137	1.39E-02	−0.208	5.81E-01	−0.554	2.10E-01	−0.674	1.30E-01
**MMP1**	0.063	9.27E-01	−0.574	4.15E-01	0.584	4.07E-01	−0.402	5.63E-01	0.208	7.67E-01	−0.457	5.15E-01
**MMP2**	1.614	5.52E-06	0.811	2.11E-02	1.07	3.32E-03	1.748	2.51E-06	1.215	1.10E-03	0.761	2.95E-02
**MMP3**	−0.661	2.75E-01	−0.628	3.15E-01	−0.989	1.19E-01	0.13	8.25E-01	0.011	9.86E-01	−0.28	6.52E-01
**MMP7**	−1.308	3.44E-01	−4.488	1.74E-04	−5.816	6.01E-06	−2.72	6.37E-02	−4.9	6.10E-05	−4.982	4.95E-05
**MMP9**	2.04	5.15E-02	1.651	3.21E-01	2.523	5.81E-03	1.966	5.93E-02	0.902	2.92E-01	1.04	2.25E-01
**MMP11**	3.986	9.23E-05	1.9	1.99E-02	1.401	3.04E-02	4.162	6.30E-05	2.464	4.48E-04	1.536	1.86E-02
**MMP12**	1.09	5.60E-01	0.861	7.06E-01	2.737	2.37E-01	0.806	6.65E-01	0.676	7.67E-01	1.148	6.16E-01
**MMP14**	3.188	1.10E-07	1.89	6.15E-04	1.895	6.01E-04	3.588	3.11E-08	2.166	1.40E-04	1.196	2.04E-02
**MMP15**	0.898	3.99E-02	0.046	9.21E-01	0.225	6.28E-01	0.985	2.66E-02	0.572	2.24E-01	0.376	4.19E-01
**MMP16**	1.878	7.59E-05	0.328	6.04E-01	0.607	3.40E-01	1.7	1.80E-04	0.946	1.43E-01	0.799	2.13E-01
**MMP19**	0.888	4.37E-02	0.795	4.31E-02	0.459	2.30E-01	0.474	2.51E-01	1.053	9.17E-03	1.124	5.81E-03
**MMP23B**	1.292	5.96E-04	0.07	8.80E-01	1.063	2.93E-02	1.346	4.30E-04	1.06	2.98E-02	−0.024	9.59E-01
**MMP25**	−0.66	1.63E-01	−0.711	4.07E-02	−0.705	4.22E-02	−0.793	9.95E-02	−0.637	6.46E-02	−0.761	2.95E-02
***TIMP1***	1.872	5.64E-08	1.243	7.30E-03	1.413	2.76E-03	1.835	6.97E-08	1.542	1.29E-03	0.942	3.62E-02
***TIMP2***	1.892	8.17E-08	0.969	1.63E-02	1.467	6.45E-04	2.03	3.85E-08	1.207	3.65E-03	0.346	3.67E-01
***A2M***	0.067	8.63E-01	0.482	3.89E-01	0.069	9.01E-01	−0.231	5.57E-01	0.083	8.81E-01	−0.238	6.69E-01

*Biopsy specimens were collected over 133 days postwound and combined in 3 wound-healing stages, early, mid, and late, for analysis. Reported values are the log_2_ fold change. Significant fold change (>1.3 FC and/or *P* < .05) in at least 1 time point. FC indicates fold change.
